# Extensional and shear rheology of okra polysaccharides in the presence of artificial saliva

**DOI:** 10.1038/s41538-018-0029-1

**Published:** 2018-11-20

**Authors:** Bo Yuan, Christos Ritzoulis, Jianshe Chen

**Affiliations:** 1 School of Food Science and Biotechnology, Zhejiang Gongshang University, Xiasha, Hangzhou, Zhejiang, 310018 China; 2 Department of Food Technology, ATEI of Thessaloniki, 57400 Thessaloniki, Greece

**Keywords:** Rheology, Biological physics, Biopolymers

## Abstract

Extensional and shear viscosities were studied for mixtures comprising artificial saliva and okra mucilage, the latter acting as a model thick-liquid food. These experiments aimed to obtain information on the flow-behavior information of the systems, underpinning the texture sensation of foods as perceived by dysphagic and xerostomic populations. Mixing okra mucilage with artificial saliva dramatically increases the shear viscosity of artificial saliva throughout the studied ranges of concentrations and deformation rates. Particle tracking does not suggest direct interactions between the components of artificial saliva and of okra mucilage. The rheology of the okra polymer (OP)–artificial saliva (AS) mixture is dominated by its extensional viscosity: Trouton ratios are in the order of tens to hundreds, while they decrease with increasing okra-mucilage concentration; this highlighs the dominance of the extensional rheology and the increasing importance of the shear mechanics with increasing okra content. The relaxation times and extensional moduli are also reported for the systems under study. Extensional and shear flows are of equal importance concerning the elastic behavior.

## Texture sensation: rheological data guide artificial saliva formulation

Artificial saliva is usually formulated to help people with dysphagia or xerostomia. Their mouth feels of food textures are largely dependent on the flow behaviors of the food–artificial saliva mixtures. Christos Ritzoulis and coworkers from Zhejiang Gongshang University now studied the rheological and structural properties of artificial saliva when mixed with okra mucilage. They found the shear viscosity and storage modulus of the artificial saliva were much higher in the presence of okra polysaccharide, suggesting the formation of a macromolecular-interacting network. Moreover, it was found the overall rheological behaviors of the mixtures were always dominated by the extensional viscosity, though the shear behavior became more and more obvious with the increasing amount of okra polymer. Together with the data obtained with real saliva, these on artificial saliva could guide the future formulation of artificial saliva.

## Introduction

In the oral cavity, saliva, produced by the salivary glands, aids mastication by means of helping the transportation of food, while it provides the necessary lubrication between the hard and soft tooth tissues. Saliva has a number of essential functions in food oral processing.^1^ Changes in viscosity, lubrication and friction, formation of aggregates and precipitates, enzymatic breakdown, destabilization of colloidal systems, and/or release of flavor compounds, will all contribute towards overall food perception.^2,3^ Salivary pH can range from 5.3 to 7.8, with a daily flow in healthy individuals between approximately 1 and 1.5 L.^4^ Although it consists of about 99.5% water and 0.5% solids, its main components are the mucins.^5^ Chemical composition of natural saliva is very complex and is influenced by a number of factors such as age, health status, or diet.^4^ There are many components of real saliva that serve various complex biological processes, hence it is difficult to produce artificial saliva that fully matches the composition of natural saliva.

Xerostomia is a condition characterized by the reduction or by the disappearance of salivary flow, and can lead to severe dryness of the oral cavity.^6^ Patients with xerostomia may experience difficulty in speaking, eating, and swallowing^7^, while this condition can favor polydipsia.^6^ Xerostomic people usually use salivary substitutes (oral lubricants). Among saliva substitutes, mouth-rinse solutions containing sodium carboxymethylcellulose (CMC) or animal mucins have been extensively used.^8^

Abelmoschus esculentus L. (okra) is a plant of the Malvacae family. It is an integral part in the diet of Africa, India, parts of Europe and America, and other countries, with a worldwide production grossing value estimated by FAO to be over $3 billion in 2014.^9^ The characteristic thick and particularly slimy texture of okra water extracts is attributed to its polysaccharide content, and is of primary technological interest for food and non-food applications.^10^ Such extracts can be used as natural food-grade emulsifiers^11^ or thickeners and emulsion stabilizers,^12^ suggesting that okra can be a promising source of texture modifiers for complex food matrices.

The principal polysaccharides of okra mucilages are type I partially methylated and/or acetylated rhamnogalacturonnans, bearing relatively small galactosyl-residue side branches.^13,14^ Ghori et al.^15^ report a maximum solubility of okra mucilages at pH 7.4, suggesting maximum chain unfolding close to neutral pH. The intrinsic viscosity of okra polysaccharides is largely dependent on the extraction protocol,^16,17^ hence the structural attributes of the supermolecular structures are dependent on the extraction sequence as well.^18^ The extracts from okra are also particular due to their viscoelastic component.^19^ Senghamparn et al. ^20^ report that their shear elastic response can be dependent on the polysaccharide acetylation content, hence the inter-macromolecular interactions. This is in line with the recent finding that okra polysacchrides exist in hexameric self-assembled forms in aqueous solutions.^21^ Their rheology is dominated by extensional viscosity, with the Trouton ratio values are in the order of tens to hundreds at pH 4 and pH 7.^22^

Chan,^23^ and Chen et al.,^24^ report that the fracture and breaking of a food, as well as the yield and flow of a food, all depend highly on its rheological properties. Moreover, the sensations elicited during the comsumption of a food are more related to the properties of food–saliva mixtures rather than to the intrinsic properties of foods.^2^ Therefore, understanding the oral rheology of such hydrocolloids, and, by extrapolation, understanding the physico-chemical basis of their mouthfeel, requires information on the rheology of their mixtures with saliva. Information on the rheological behavior of food–saliva mixtures is instrumental in linking rheological proprties of foods to sensory data.^25^ This work aims in providing a detailed rheological and structural characterization of mixed okra mucilage–artificial saliva mixtures involving extensional and shear rheology, particle tracking analysis, and interfacial tension measurements. The results are compared to new data on extensional viscosity of okra polysaccharide and human oral cavity saliva mixtures and okra polysaccharide alone, as to come out with a self-consistent description of the systems that are responsible for the manifestation of mouthfeel of okra mucilage and of similar hydrocolloids, and to give information prerequisite to understanding the action and improving artificial saliva formulations. Taken together with data on the elongational and shear viscosity of hydrocolloid mixtures with real saliva, design prerequisites can be established for new artificial saliva formulations, and well as for the optimization of existing xerostomic products.

## Results and Discussion

### Shear rheology

**Fig. 1:**
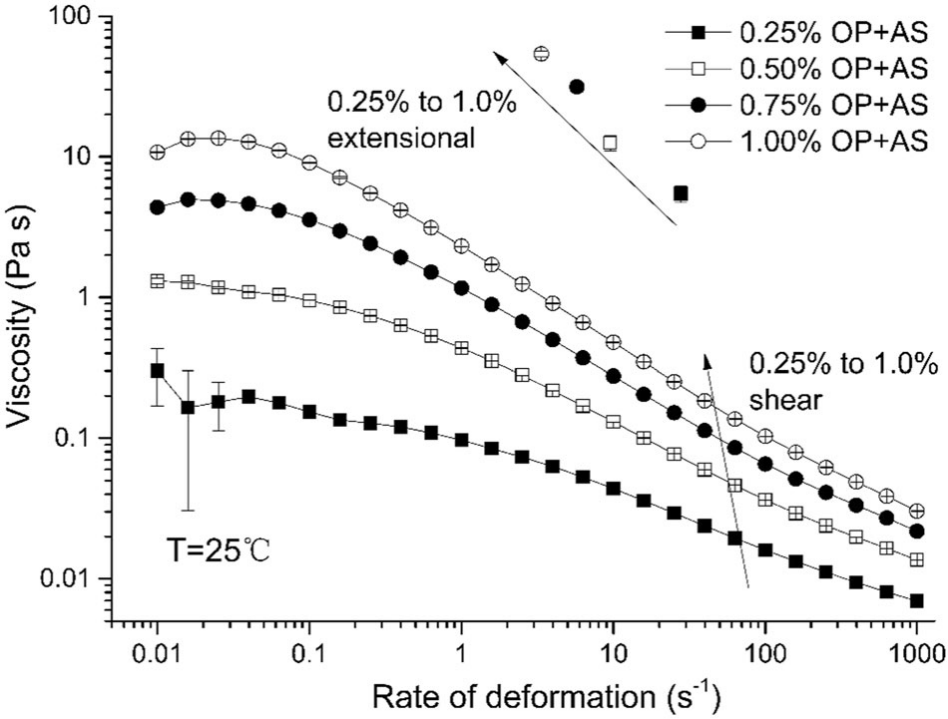
Plots of shear viscosities vs shear strain for a series of okra polymer (OP) solutions mixed with artificial saliva at 1:1 v/v ratio at 25 °C. Points at top: Extensional viscosities vs rates at 25 °C for okra polysaccharide (OP) solutions mixed with artificial saliva at 1:1 v/v ratio (concentrations shown increase in increments of 0.25%)

Commercial artificial saliva was mixed with an equal volume of buffers containing different amounts of aqueous okra polymer (OP) solution, were studied as to provide an impression of the fate of liquid food in the oral cavity in the presence of salivary substitutes (a typical scenario in xerostomic people). Figure 1 shows the shear viscosity profile of all concentrations of OP in mixtures with artificial saliva (AS) over five magnitudes of shear rate (0.01 s^−1^ to 1000 s^−1^). All examined samples show shear-thinning. Artificial saliva, diluted 1:1 with tris buffer, shows a high-viscosity low-shear plateau (about 0.12 Pa s at 0.01 s^−1^) decreasing in viscosity (down to a value close to that of pure water, 10^−3^ Pa s) as shear rate increases. Increase in okra mucilage up to a final content of 0.25% results in viscosity increase (0.01 Pa s at 1000 s^−1^). This is in line with what is expected from a polymer network consisting of non-interacting units. Further increases in OP concentration result in further viscosity increases throughout the range of the shear rates under study. The highest viscosity is attained at 1.0% OP–AS, with a value of about 13 Pa s at 0.01 s^−1^. This is within the order of size with that attained for a 1:1 mixture of 1.0% OP and human saliva.^26^ A similar comparison also exists for the high-strain rate regime.

**Fig. 2:**
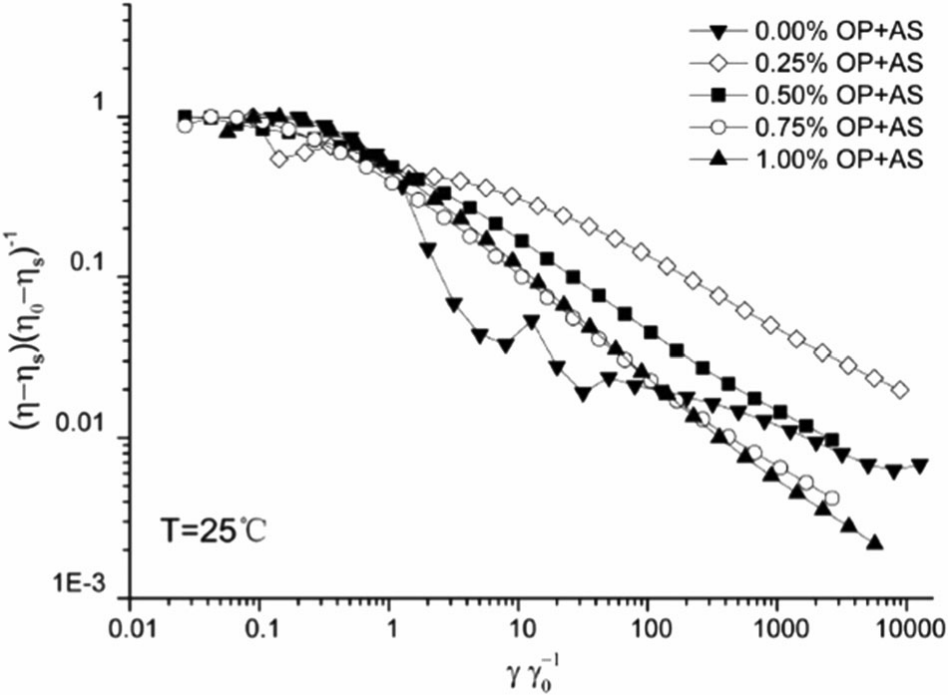
Master shear-thinning curves for okra polymer (OP) solutions mixed at 1:1 v/v ratio with artificial saliva. Concentrations are provided on the plot

Information on the effect of the OP addition into the artificial saliva fluid can be inferred via the normalization of the shear flow curves with the solvent viscosity at an arbitrary shear rate by means of overlaying the relative shear viscosities (*η* − *η*_*s*_) (*η*_0_ − *η*_s_)^−1^ vs the ratio of shear rate *γ* over the shear rate that induces reduction in the solvent-exempt viscosity by half *γ*_1/2_ (where *η* is the shear viscosity, *η*_0_ is the low-strain maximum viscosity, and *η*_s_ is the solvent’s viscosity^27^). Considering that the polymers of OP and artificial saliva share structural characteristics (they contain polysaccharides), and that they interact in similar manner, the above curves should overlay. Such an overly is noticed indeed (Fig. 2), but deviations exist for the plots of the lower OP concentrations up to an OP content of 0.25%. This implies that similar structural attributes and interactions do exist within this concentration regime.

**Fig. 3:**
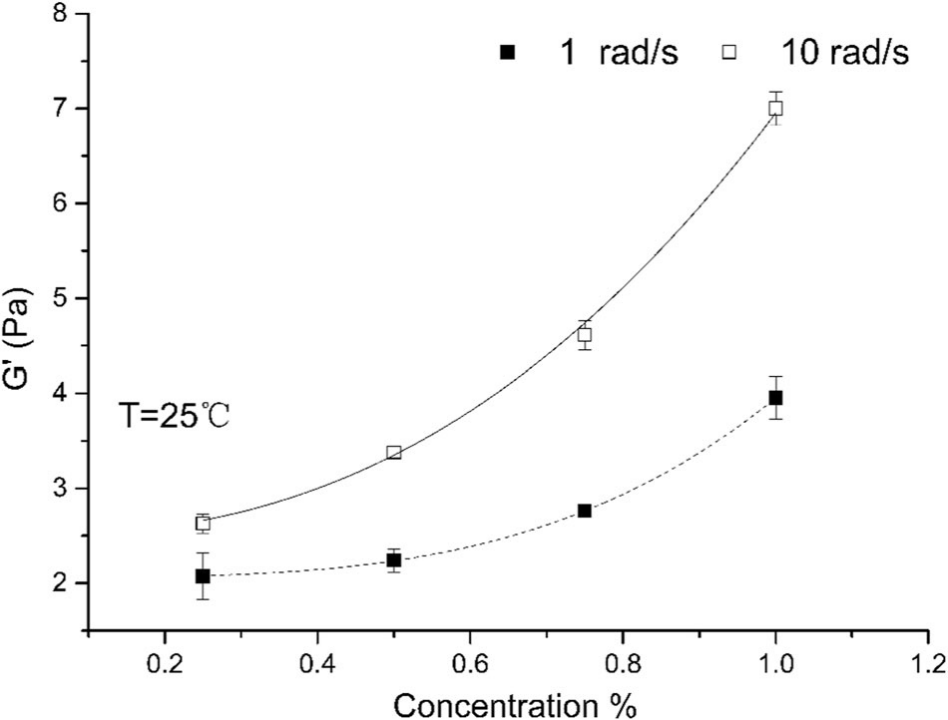
Comparison of small-deformation storage (*G*′) moduli vs final concentration of okra polymer at an angular velocity frequency of 10 rad s^−1^ (top) and 1 rad s^−1^ (bottom) for okra polymer (OP) solutions mixed at 1:1 v/v ratio with artificial saliva at 25 °C. Measurements were carried out at maximal strain value of 0.1%, within the linear viscoelastic region

The storage moduli are higher than the loss moduli throughout the studied angular velocity range at all concentrations, including artificial saliva diluted 1:1 with buffer (data not shown). In Fig. 3, the *G*’ values have been plotted against OP concentration at 1 rad s^−1^ and at 10 rad s^−1^. This serves as to assess the effect of okra mucilage on the elastic properties of the artificial saliva–okra polymer mixtures. Although hydrocolloids do pre-exist in the sample, their constant and low concentration allows them to be treated as a background for this set of measurements. Storage moduli increase in a monotonic fashion in both two angular velocities, with higher values observed at the higher angular velocity 10 rad s^−1^. This non-linear increase with concentration suggests that the semi-dilute threshold is below the concentrations under study. The data can be readily fitted into a power law equations *G*′ = 2.06 + 1.90 *C*^3.4^ for 1 rad s^−1^and *G*′ = 2.54 + 4.45 *C*^2.6^ for 10 rad s^−1^.

### Particle size analysis

The samples under study are made up of two liquids with discrete macromolecular populations: artificial saliva comprising polysaccharides, and okra polymers comprising mostly of rhamnogalacturonnans.^13^ In order to investigate the fate of these macromolecular populations in the mixtures under examination, the size distributions were studied using a dedicated particle-tracking analyzer that is able to detect and quantify particles of different orders of size within the same sample. As the samples under investigation were essentially diluted, information involving packing or excluded volume interactions may be partially compromised, while information on aggregates formed from direct and irreversible interactions will not be lost.

**Fig. 4:**
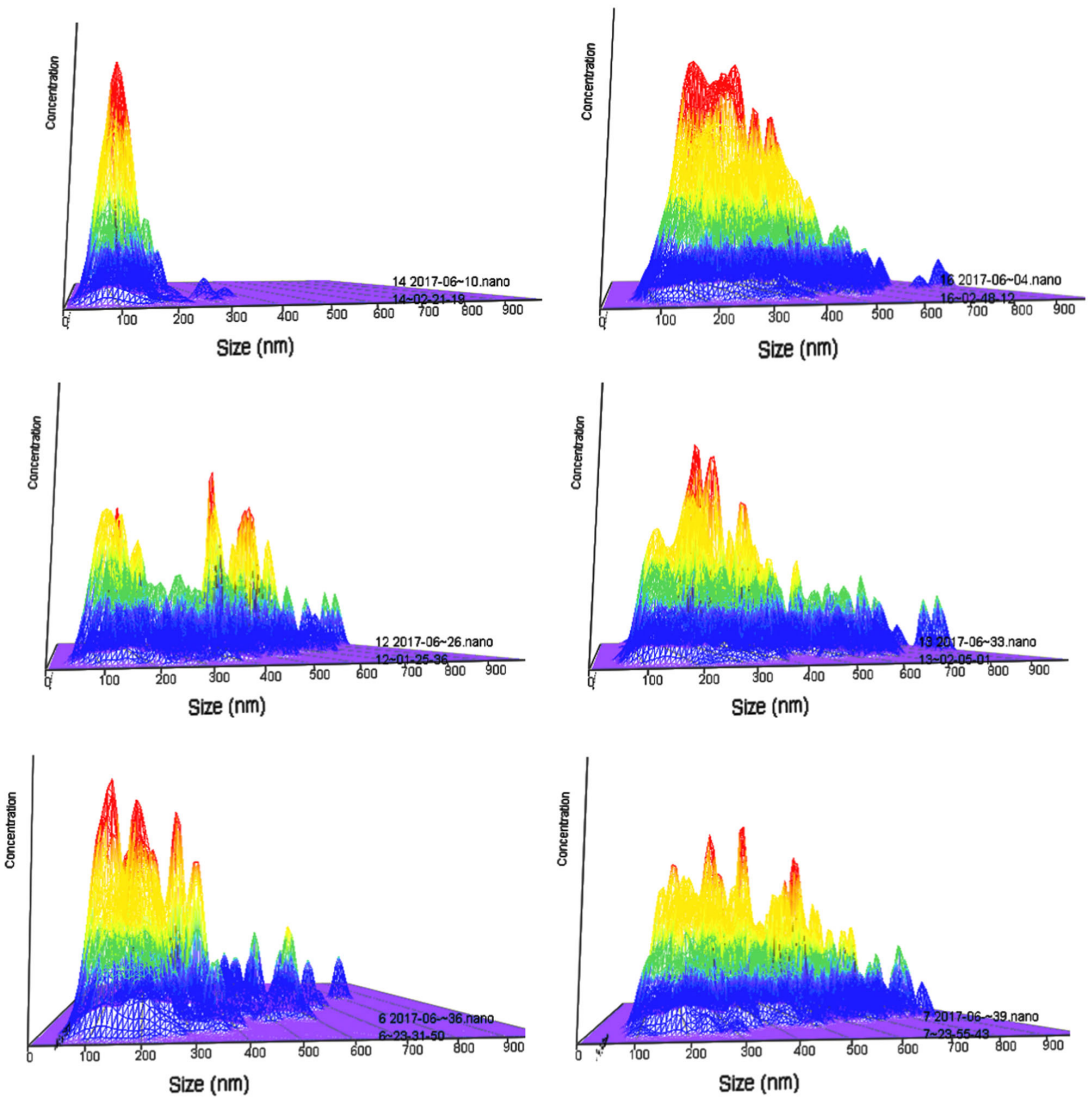
Size distributions for particles of okra polysaccharide (OP) dispersions mixed at 1:1 v/v ratio with artificial saliva and diluted down to an OP content of 0.05% at 25 °C (x-axis: size, y-axis: intensity and z-axis: concentration). Top left to bottom right: **a** artificial saliva (no OP), **b** OP (diluted with plain buffer, no artificial saliva), **c** 0.25% OP in artificial saliva, **d** 0.5% OP in artificial saliva, **e** 0.75% OP in artificial saliva, and **f** 1.0% OP in artificial saliva

Figure 4 shows the evolution of particle-size distributions during the mixing of artificial saliva with increasing concentrations of OP. The three-dimensional graphs show sizes (x-axis), concentration (y-axis) and intensity (z-axis). The upper left plot shows the sizing results for diluted AS in the absence of OP, any macroscopical populations appear to exist in entities of about 100 nm, with few, if any, particles ranging over 150 nm (top left). The upper right plot shows the concentration vs size plot of OP in the absence of artificial saliva; This is clearly a polydisperse polymer mixture, which is in agreement with this group’s microcalorimetric report of the multitude of metastable supermolecular structures that OP assumes.^21^ The most abundant populations are within the size of 100–200 nm, while structures of up to 600 nm can be found in fairly large amounts. Increase in the content of OP in artificial saliva results in an increase of the populations above 300 nm and up to 600 nm, as per OP. Then this trend continues (mid-left, then right, then bottom left to bottom right for increasing OP content in artificial saliva).

### Extensional rheology

Shear rheometry is limited to the study of a material’s reaction to shear forces; that is, particles contained in a fluid are aligned by sliding over each other while maintaining similar interparticle distances. In reality, however, the applied stresses in a fluid during oral-processing fluids undergo expansion or compression. That sort of extensional flow may instigate much higher deformations at the colloidal scale in comparison with shear deformation.^28^ It is accepted that oral processing (mastication and swallowing) involves both shear and extensional deformation components.^1,24^ Furthermore, the human threshold in detecting extensional viscosity is lower than that of the shear viscosity.^29^ That hints that human has a greater-discriminatory capacity in perceiving extensional viscosity, yet this is largely understudied. Therefore, understanding of the behavior of artificial saliva–hydrocolloid materials under elongational deformations can add a new, largely unexplored, dimension to the description of the oral processing mechanics. Under this rationale, the samples studied with shear rheometry in the previous sections were now subjected to an examination of their reaction to elongational deformations.

**Fig. 5:**
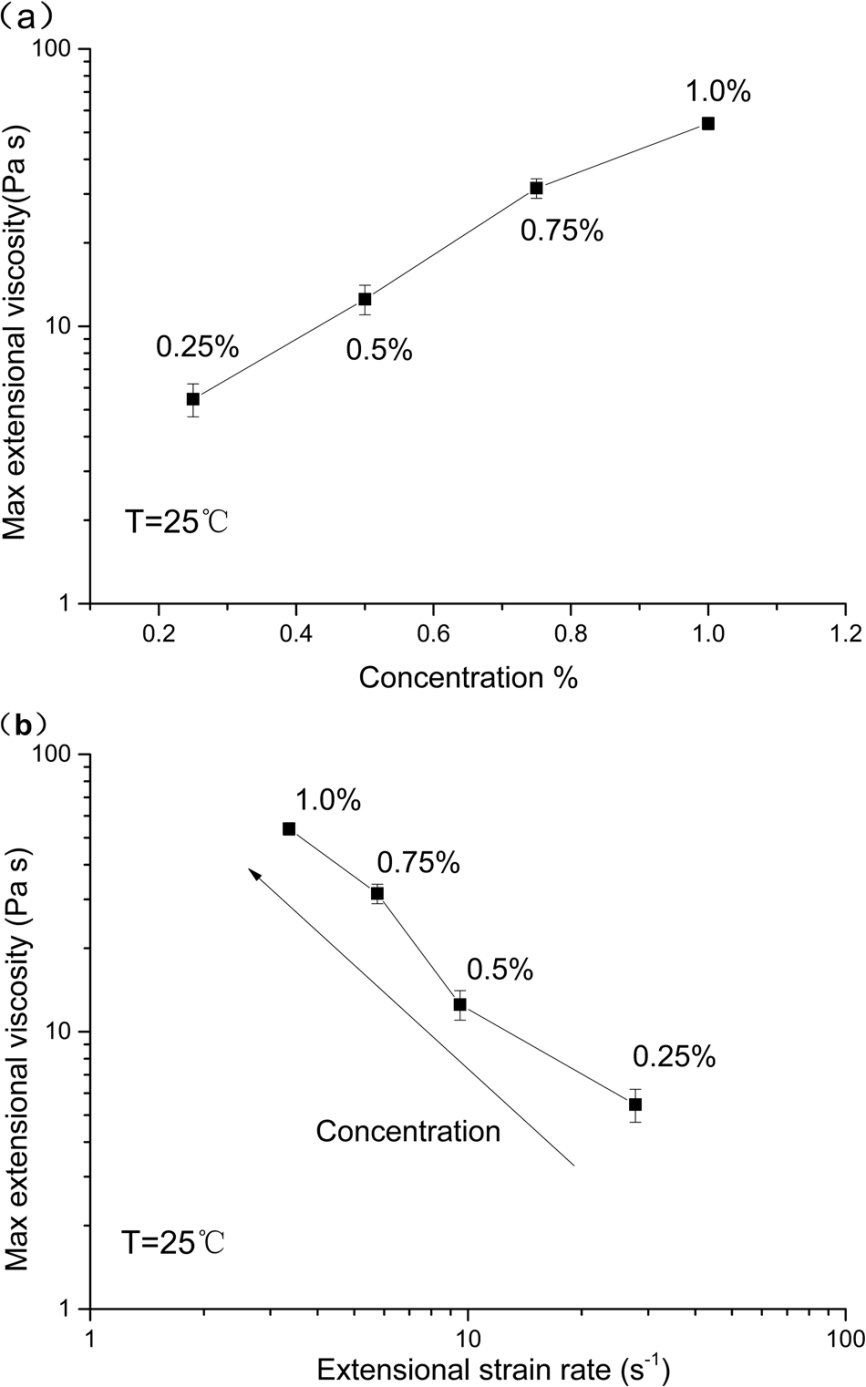
Maximum recorded extensional viscosity for okra polymer (OP) solutions mixed with artificial saliva at 1:1 v/v ratio at 25 °C vs **a** the final okra polymer concentrations and **b** extensional strain rate

Figure 5 depicts the maximum extensional viscosity plotted against concentration (top part of the plot) and the maximum extensional viscosity plotted against the relevant extensional strain rate (bottom part of the plot). Lower concentrations, being less resistant to flow, show higher-extensional rate (one point per concentration, with concentrations increasing from right to left). As the device used has no direct control of stress or extensional rate, only the maximum viscosity is attained for each concentration, along with its equivalent extensional strain rate. These values increase from about 5 Pa s at 0.25% OP to about 55 Pa s at 1% OP.

**Fig. 6:**
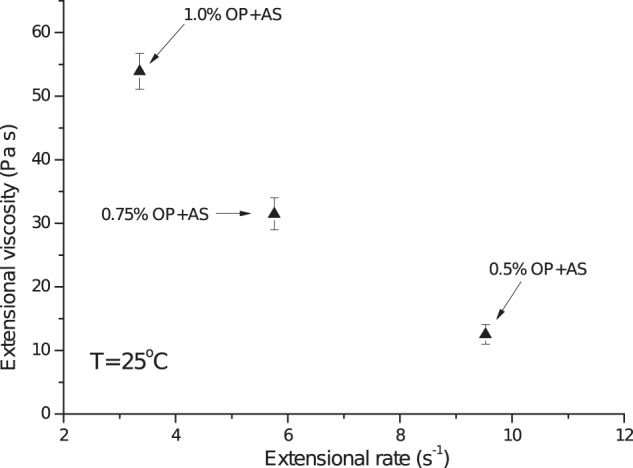
Maximum extensional viscosities vs extensional rates at 25 °C for 1:1 v/v of artificial saliva (AS) with okra polymer (OP) solutions

Figure 6 depicts the maximum extensional viscosities vs the extensional rates obtained for the mixtures under study (1:1 of artificial saliva with OP solutions). When compared to this group’s existing data on real saliva–OP mixtures recently studied under similar conditions^26^ (1:1 of human oral cavity saliva with OP solutions), one can see that the extensional viscosities vs. rates are broadly matching.

Okra gum^22,30^ as well as other hydrocolloids are reported to possess fairly high values of extensional viscosity^31,32^. Figure 1 also plots the extensional viscosities of the samples against the extensional rate (please note that the concentrations increase from right to left); for comparison purposes, this set of data is overlayed on the shear rheological data of the previous paragraphs at the equivalent shear strain rates. For all solutions under study, the extensional viscosity values are more or less one order of size higher than the equivalent shear ones. The rheology of hydrocolloid–artificial saliva mixtures is thus dominated by its extensional behavior rather than the shear one. This is important when considering the design of formulations for xerostomia. The Trouton ratios for OP–AS mixtures drop from 270 at 0.25% OP to 75 at 1% OP (Table 1). That confirms again the dominance of extensional viscosity as opposed to the shear one regarding AS–OP fluid mechanics. These values are appreciably higher than the ones reported for other biopolymers such as milk proteins and potato starches.^23^ The relative importance of the extensional rheology is higher at low concentrations, while it diminishes at higher concentrations. This suggests that, at higher concentrations, the closer packing of macromolecules brings about stronger resistance to shear forces; that in turn increases the shear component’s importance. Still, the extensional component is dominant even at these concentrations.

**Table 1:** Trouton ratio values for a series of okra polymer solutions mixed with artificial saliva at 1:1 v/v ratio

Concentration (%)	0.25	0.5	0.75	1.0
Trouton ratio	269 ± 12	95 ± 4	92 ± 5	75 ± 3

As a conclusion, one may state the following: Incorporation of okra polysaccharide (OP) solutions in a commercial AS formulation increases the shear viscosity of the systems throughout the shear-strain range under study from 0.12 Pas to 13 Pa s in the low strain rates (0.01 s^−1^), while storage moduli increase in a monotonic fashion from 2 to 8 Pa in the concentration regime between 0.25 and 1% of OP. These suggest a network of interacting macromolecules. Extensional viscosity dominates the overall behavior of okra mucilage–saliva mixtures. Maximum extensional viscosity values increase from about 5 Pa s at 0.25% OP to about 55 Pa s at 1% OP. OP–AS mixtures are predominantly elastic in character. Trouton ratio values are in the order of hundreds for the range of concentrations for the deformation rates under study. Extensional and shear flows are equally important concerning the OP–AS elastic behavior. Such information can be used in conjunction with data from real saliva as to provide further guidelines for the optimization of artificial saliva formulations.

## Methods

### Materials

Type 1 ultrapure water, used in all experiments, was obtained from a Millipore Direct-Q 5UV apparatus (Merck, Darmstadt, Germany). Tris buffer was purchased from AMRESCO (Solon, OH) and sodium hydroxide from Xilong (Xilong Scientific Co, Shantou, China). A commercial artificial saliva product (Disaccharide Dry Ease Gum Gargle, Shandong Saikesaisi Co., Ltd, Shandong, China) was used for the experiments.

### Okra extraction and purification

Okra fruit ds were purchased from the local super market. Their pods were cleaned, the stem and the seeds were separated, while the rest of the fruit was cut into ~5 mm-thick slices. Hundred grams of these slices was immersed into ultrapure water set at pH 6.4 ± 0.2 at 55 °C, and left under magnetic stirring for 90 min. This liquid was then separated from the solids and was condensed down to approximately 100 mL using a rotary evaporator operating at 60 °C under vacuum. This condensate was centrifuged at 5000 g for 30 min at 25 °C, and was then dialyzed (MWCO 3500 Da) against a large volume of ultrapure water for 6 h, in order to remove the smaller molecules. The dialysis process was repeated three times with fresh ultrapure water. The resulting viscous liquid was then freeze-dried using a Scientz-10N freeze dryer (Ningbo Scientz Biotechnology co., ltd., Ningbo, China), and was then packed in sealed containers and stored in fridge for further use.

### Sample preparation

Okra powder, as prepared above (0.1–2 g, depending on the measurement), was put into containers, and 100 mL of a 10 mM solution of Tris buffer were subsequently added in them; the containers were then sealed and were left stirring overnight in order to ensure complete solubilization. Solutions of 1:1 v/v okra polysaccharide (OP) + AS were prepared, ranging from 0.00 to 1.00% final OP content. All preparations and experiments were conducted at a controlled temperature of 25 °C. Distinct samples were used for the measurements. Experiments were held at 25 °C, a temperature found to be an approximation for the oral processing of many foods.^33^

### Nanoparticle tracking analysis

The sizes and the size distributions of all macromolecular populations were quantified with a Malvern NanoSight NS300 nanoparticle-tracking analyzer (Malvern instruments, Worcestershire, UK). Samples were passed through a 0.45 μm syringe filter before their insertion into the device’s measuring chamber. The conditions were: continuous phase viscosity 1 mPa s and temperature 25 °C. After successive dilutions and trial and error measurements using the appropriate buffers, results were recorded for 1:1 v/v okra polysaccharide (OP)–AS solutions, at a 0.05% final OP content. Measurements were taken for 60 s per run. Measurements were performed in triplicate.

### Shear rheology

A TA model Discovery HR-2 controlled strain-rate-bench rheometer (TA, New Castle, DE) was used for all shear rheometry experiments. The device was equipped with a 40 mm cone and plate geometry (angle of 2.008°), which was rotating on top of a smooth aluminium Peltier-thermostated surface (25 °C); the two surfaces were separated by a 56 μm-set gap; a total of 0.6 ml of fluid sample was used. The samples underwent shear-sweep tests at a shear rate range of 0.01–1000 s^−1^. The intraoral fluids temperature may range between 5–55 °C during mastication,^34^ as the natural temperature of human saliva can rise or drop during oral processing due to its difference with the initial temperature of the food. In this work a temperature of 25 °C was chosen for the mixture of AS with a model cold-ambient hydrocolloid.^33^ Measurements were repeated in duplicate.

### Extensional rheology

The extensional rheology of the samples has been measured at 25 °C using a specialized commercial filament-breakup device (CaBER1 extensional rheometer, Thermo Haake GmbH, Karlsruhe, Germany). This method’s principle is to measure the diameter of the mid-point of a filament, as is stretched between two plates. The latter have a diameter of 6 mm with a 3 mm initial gap. The samples were initially loaded between the plates using a 1 mL pipette, and were subsequently stretched linearly for 50 milliseconds unitl reaching a final gap of 8.91 mm, with a stretching velocity of 118.2 mm s^−1^. After this point surface-tension causes the filament to thin down, the rate of thinning being a function of the surface tension of and the fluid extensional viscosity.^35,36^ The diameter of the filament was under continuous measurement using a laser beam at mid-point of the final gap height (*D*_mid_), from the initial diameter *D*_0_ at time *t*_0_ (when the top plate starts moving) up to the filament break-up time, *t*_b_ (when the filament breaks). The measurements were repeated ten times for each sample, and the means were taken for further analysis.

The apparent extensional viscosity *η*_E_ of the samples can be calculated from the thinning behavior of the filament after the top plate has reached the final gap, using the equation developed by McKinley and Tripathi^37,36^

1 $${\eta _{\mathrm{E}} = \left( {{\mathrm{2}}X - {\mathrm{1}}} \right)\frac{\sigma }{{\frac{{ - dD_{\mathrm{mid}}\left( t \right)}}{{dt}}}}}$$

Where *σ* is the surface tension of the fluid, *D*_mid_ is the mid-filament diameter and *X* is a geometry coefficient, which takes into account the shape of the filament during thinning due to inertial and gravitational effects.^35,37^ It was shown experimentally that a value of *X* = 0.7127 is an appropriate value for highly viscous fluids.^37^

The approximate derivative of the mid-filament diameter can be numerically obtained from the adjacent values at a given time,^35^ where *i* is the current time:

2 $${\frac{{dD_{\mathrm{mid}}\left( t \right)}}{{dt}} = \frac{{D_{\mathrm{mid}}\left( {t_i} \right) - D_{\mathrm{mid}}\left( {t_{i - {\mathrm{1}}}} \right)}}{{t_i - t_{i - {\mathrm{1}}}}}}$$

The Hencky strain of the thinning filament can be calculated using the following equation:^35,36^

3 $${\varepsilon = {\mathrm{2ln}}\left( {\frac{{D_{\mathrm{0}}}}{{D_{\mathrm{mid}}\left( t \right)}}} \right)}$$

Fitted models can be used as to obtain data on the extensional elastic moduli and on the relaxation times. An upper convected Maxwell equation, which is appropriate for elastic systems, was found to yield the best fits at all concentrations under study:

4 $${\frac{D}{{D_o}} = \left( {\frac{{{\mathrm{GD}}_o}}{\sigma }} \right)^{\frac{{\mathrm{1}}}{{\mathrm{3}}}}e^{\frac{{ - t}}{{{\mathrm{3}}\lambda }}}}$$

Here *G* is the modulus, *D*_o_ is the initial filament diameter, and *λ* is the relaxation time. Surface tension is measured and entered as an independent variable in Equation (4) as to derive parameters such as the moduli and the relaxation times.

Error bars stand for standard deviation.

### Surface tension measurements

Surface tensions were measured with a DWK tensiometer (Harke, Beijing, China) equipped with a Wilhelmy plate. The solutions’ surface tensions were monitored until equilibrium. Temperature was controlled at 25 °C. At least three replicates were performed for each test.

## Data Availability

The data that support the findings of this study are available from the corresponding author upon reasonable request.
